# Time Trends and Predictors of Abnormal Postoperative Body Temperature in Infants Transported to the Intensive Care Unit

**DOI:** 10.1155/2016/7318137

**Published:** 2016-09-29

**Authors:** Hedwig Schroeck, Angela K. Lyden, Wendy L. Benedict, Satya Krishna Ramachandran

**Affiliations:** ^1^Department of Anesthesiology, Dartmouth-Hitchcock Medical Center, 1 Medical Center Drive, Lebanon, NH 03756, USA; ^2^Department of Anesthesiology, University of Michigan Health System, 1500 E Medical Center Drive, Ann Arbor, MI 48109, USA; ^3^Department of Anesthesia, Critical Care and Pain Medicine, Beth Israel Deaconess Medical Center, 330 Brookline Avenue, Boston, MA 02215, USA

## Abstract

*Background*. Despite increasing adoption of active warming methods over the recent years, little is known about the effectiveness of these interventions on the occurrence of abnormal postoperative temperatures in sick infants.* Methods*. Preoperative and postoperative temperature readings, patient characteristics, and procedural factors of critically ill infants at a single institution were retrieved retrospectively from June 2006 until May 2014. The primary endpoints were the incidence and trend of postoperative hypothermia and hyperthermia on arrival at the intensive care units. Univariate and adjusted analyses were performed to identify factors independently associated with abnormal postoperative temperatures.* Results*. 2,350 cases were included. 82% were normothermic postoperatively, while hypothermia and hyperthermia each occurred in 9% of cases. During the study period, hypothermia decreased from 24% to 2% (*p* < 0.0001) while hyperthermia remained unchanged (13% in 2006, 8% in 2014, *p* = 0.357). Factors independently associated with hypothermia were higher ASA status (*p* = 0.02), lack of intraoperative convective warming (*p* < 0.001) and procedure date before 2010 (*p* < 0.001). Independent associations for postoperative hyperthermia included lower body weight (*p* = 0.01) and procedure date before 2010 (*p* < 0.001).* Conclusions*. We report an increase in postoperative normothermia rates in critically ill infants from 2006 until 2014. Careful monitoring to avoid overcorrection and hyperthermia is recommended.

## 1. Background

Postoperative hypothermia is a recognized complication of anesthesia and surgery in children with many deleterious side effects, including altered coagulation, increased risk of surgical site infections, cardiac dysrhythmias, and apnea [1, 2]. The normal physiologic response to hypothermia drives metabolism of fat and shivering. These responses are associated with increased oxygen requirements and increases in cardiac output, which are not well tolerated by critically ill neonates and infants [3–5]. Therefore, perioperative temperature management tends to focus on preventing hypothermia [6]. This is typically accomplished by a combination of techniques, including use of infrared lights, convective warming blankets, and plastic wraps and covers of exposed skin surface (e.g., the infant's head). To prevent cooling during transfer, neonates are usually covered with warm blankets to minimize exposed skin. Extremely vulnerable patients may also be transported in closed incubators during patient transfer to the neonatal intensive care unit (NICU), but this is not routine practice in the postsurgical setting and data regarding the efficiency are lacking.

Our institution implemented aggressive strategies for the prevention of postoperative hypothermia including the widespread use of active intraoperative convective warming and introduction of closed-top incubators for postoperative infant transports in the year 2010, providing a unique opportunity to study the impact of these measures. A local audit of posttransfer hypothermia in sick infants revealed an unexpectedly high incidence of postoperative hyperthermia after implementation of these quality improvement efforts. Therefore, this investigation was done to determine the frequency of postoperative hypothermia <36°C and postoperative hyperthermia >37.5°C in all infants returning to the intensive care unit (ICU) from the operating room over a 9-year time period. The secondary aim was to determine the independent factors associated with postoperative hypothermia and hyperthermia in these critically ill infants.

## 2. Methods

A data query from our single-institution Anesthesia Information Management System (AIMS) database (GE Healthcare, Waukesha, WI) was performed with approval from our institutional review board (HUM00061293). Patient/guardian consent was waived secondary to the retrospective nature and retrieval of deidentified data. All ICU infants (aged 0–365 days) who were transferred to the neonatal or pediatric intensive care unit immediately postoperatively between June 1, 2006, and May 31, 2014, were identified. This AIMS database has been utilized in several publications related to pediatric perioperative outcomes in the past [7, 8].

The following independent temperature related variables were collected from discrete data fields in the AIMS record: preoperative temperature, site of intraoperative temperature monitoring (nasal, oral, oropharyngeal, esophageal, bladder, rectal, or skin), highest intraoperative temperature (defined as the highest identical value measured at three consecutive time points to minimize measurement artifacts), intraoperative temperature measured at completion of surgical procedure, postoperative temperature measured immediately after transfer to the ICU, and use of a convective warming blanket. Pre- and postoperative temperature measurement in the intensive care unit is usually performed via the axillary route and then manually recorded in the anesthesia record. Intraoperative temperature data are automatically entered through monitor capture into the AIMS record. In addition, the following patient and procedural factors were collected: body weight measured in the immediate preoperative period, age in days, premature birth (<37 weeks), postdate birth (>40 weeks), ASA status (dichotomous variable for ASA class 1/2/3 versus 4/5), emergency surgical procedure, surgical specialty, and duration of surgery. Closed-top incubators for neonate and infant transfers were introduced during the year 2010. Subjects undergoing cardiac procedures and cases with missing postoperative temperature documentation were excluded.

### 2.1. Definition of Abnormal Temperature

The outcome variable was the post-ICU transfer temperature (measured on arrival in the ICU). Postoperative temperature was categorized as hypothermia (<36.0°C), normothermia (36–37.5°C), and hyperthermia (>37.5°C). While our definition of hypothermia is in accordance with the current literature [5, 9, 10], it was more difficult to identify a widely accepted threshold for postoperative hyperthermia. In the general medical literature, hyperthermia is historically and controversially defined as a temperature >37°C, whereas “postoperative fever” is more commonly defined as temperatures in excess of 38°C [11, 12]. Further, the normal core temperature may be as high as 37.5°C during the afternoon peak due to circadian temperature changes [13]. We chose a temperature of >37.5°C as our definition of hyperthermia for this study, because physiologic responses to elevated temperature are present at this level [14].

### 2.2. Statistical Analysis

Proportions of posttransfer hypothermia, normothermia, and hyperthermia were calculated for each year of the study period. In addition, the magnitude and direction of changes between preoperative and postoperative temperature were determined. Temperature recordings at any time point of less than 30°C or in excess of 43°C were deemed “implausible” and excluded from analysis. Temperature data were presented as mean ± SD. To compare the three outcome groups (postoperative hypothermia, normothermia, and hyperthermia), descriptive statistics were generated and *p* values calculated using the Pearson chi-square test for categorical variables and the *t*-test for continuous variables. A *p* value of <0.05 was accepted as significant. Trends for hypothermia and hyperthermia were described using the *r* ^2^ value of the linear regression fit lines.

Next, backward Wald parsimonious logistic regression models were developed to determine the independent associations of patient and procedural factors with the study outcomes. The following factors were specifically tested and adjusted for in the final model: weight, gender, prematurity, postdate birth, duration of anesthesia, use of intraoperative convective warming, intraoperative temperature change, procedure date before versus in/after 2010, ASA status, emergent procedure, and intraoperative temperature monitoring source. Missing data >5% was handled by performing unadjusted 2 × 2 analyses to test for outcome differences between patients with missing data and those with complete data. The size of independent associations between covariables and study outcome was evaluated by adjusted odds ratios (ORs) with 95% confidence intervals (CIs). Model goodness-of-fit was evaluated using the Omnibus Tests of Model Coefficients and the Hosmer-Lemeshow Test.

## 3. Results

Data were collected from 2747 infants transferred to the ICU immediately postoperatively during the study period. 397 cases were excluded from the final analysis due to missing (*n* = 392) or implausible (*n* = 5) postoperative temperature measurements. The remaining 2350 cases were used for analysis which included 926 subjects with more than one anesthetic.

**Figure 1:**
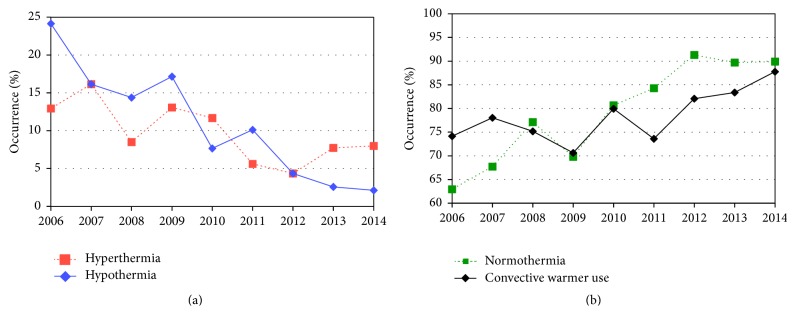
Incidence of hypothermia decreased from 2006 to 2014, whereas hyperthermia remained stable (a). Postoperative normothermia rates and use of convective warming blankets increased from 2006 to 2014 (b).

The overall mean preoperative temperature was 36.7°C ±0.53 (*n* = 1555), dropping slightly to 36.3°C ±1.5 (*n* = 1787) at surgery end and then rising slightly to 36.7°C ±0.70 (*n* = 2350) on arrival at the ICU. However, only 82% of infants (*n* = 1922, 82%) were normothermic after their procedure. Postoperative hyperthermia >37.5°C was as frequent as postoperative hypothermia <36°C (*n* = 212, 9%, and *n* = 216, 9%, resp.). Hyperthermia in excess of 38°C occurred rarely (*n* = 74, 3.1%), as did hypothermia <35°C (*n* = 26, 1.1%).  Figure 1 shows the declining incidence of abnormal postoperative temperatures over time. Hypothermia occurred in 24% of infants in 2006 and decreased to only 2% of infants in 2014 (*p* < 0.0001, Figure 1(a)), with an *r* ^2^ value of 0.896 for trend during the observation period. As shown, this trend towards normothermia started before the introduction of closed-top incubators in the year 2010. During the same time, the documented use of convective warming rose modestly from 74% to 88% (*p* = 0.012; *r* ^2^ 0.531, Figure 1(b)). Hyperthermia, after initially declining from 13% in 2006 to a low of 4% in 2012 (*p* = 0.04; *r* ^2^ 0.51), became as prevalent again in the recent years (8% in 2014; *p* = 0.357 from 2006; *p* = 0.373 from 2012).

For 1271 infants with both preoperative and end of surgery temperature data, we found that overall mean intraoperative temperature was unchanged (−0.36°C ±1.5; *p* = 0.236) for infants in the normothermia group but decreased significantly for infants who continued to be hypothermic postoperatively (−1.6°C ±1.5; *p* = 0.007). Infants who were hyperthermic postoperatively showed more thermal stability during their procedure with a mean temperature increase of 0.10°C ±1.9 until the end of surgery. For all groups, temperature rose between end of surgery and admission to the ICU (*n* = 1787; *r* ^2^ 0.54). This change was insignificant for hypothermic and normothermic infants (0.36°C ±1.5 and 0.36°C ±1.4; *p* = 0.089) but more pronounced for hyperthermic infants (1.0°C ±1.7; *p* = 0.023).

Univariate analysis was performed for each of the 3 temperature categories. Results are shown in Table 1. Of note, lower body weight and younger age were more frequently observed with both hypothermia and hyperthermia (*p* < 0.001 and *p* = 0.001, resp.). The highest intraoperative temperature measurement, use of convective warming, and longer duration of anesthesia were also more frequent with abnormal postoperative temperature (*p* < 0.001 for all). Normal postoperative temperature was more common if the procedure was performed in or after the year 2010 (*p* < 0.001).

Logistic regression analysis was performed for both the hypothermia and the hyperthermia groups (Tables 2 and 3). Postoperative hypothermia was independently associated with a higher ASA score (OR 1.7, 95% CI 1.1, 2.6; *p* = 0.02) and negatively associated with the use of convective warming (OR 0.30, 95% CI 0.2, 0.5; *p* < 0.01) or a procedure date in 2010 or later (OR 0.24, 95% CI 0.2, 0.4; *p* < 0.01). Postoperative hyperthermia was associated with temperature increases during surgery (OR 1.3, 95% CI 1.2, 1.5; *p* < 0.01) and use of convective warming (OR 2.6, 95% CI 1.1, 6.3; *p* = 0.03) and negatively associated with body weight (OR 0.85, 95% CI 0.8, 0.9; *p* < 0.01) and case date in or after 2010 (OR 0.33, 95% CI 0.2, 0.5; *p* < 0.01). All models had good fit with no evidence of multicollinearity.

## 4. Discussion

We report a significant overall increase in normothermia rates in sick infants transferred to the intensive care unit of one quaternary care hospital system during the study period. Nonetheless, abnormal temperatures continued to occur, with a trend towards increasing hyperthermia rates in recent years.

### 4.1. Predictors of Abnormal Postoperative Temperature

In the adjusted analysis, hypothermia was more likely to occur in sicker patients with higher ASA status and less likely since 2010 and when convective warming was used intraoperatively. Similarly, the risk for postoperative hyperthermia was reduced since 2010 but increased with intraoperative convective warming. Successful intraoperative warming (as manifested by an increase in body temperature by surgery end) was associated with postoperative hyperthermia, emphasizing the need for careful temperature monitoring intraoperatively to avoid overcorrection. Interestingly, while the univariate analysis suggested low body weight as a predictor for both postoperative hypothermia and hyperthermia, this association was only found for hyperthermia in the multivariate analysis. Clinically, it is well known that heat transfer in either direction, that is, heat loss or heat absorption, occurs faster in neonates and small infants [15]. Length of surgery was not independently associated with increasing body temperature, in contrast with findings from recent studies in both pediatric and adult patients [16, 17]. Depending on the exact nature of the procedure, it is plausible that longer surgeries may allow more time for temperature correction/overheating but there may be another subgroup of procedures where heating efforts cannot sufficiently counteract the ongoing heat loss, such as cases with prolonged exposure of the entire small bowel. In those specific cases, longer procedures may only lead to more heat loss.

### 4.2. Postoperative Hyperthermia

The frequent occurrence of postoperative hyperthermia in 9% of infants was unexpected, given that hypothermia is typically the overriding concern. Little evidence is found in the literature about outcome implications of postoperative hyperthermia, but hyperthermia of ≥38°C in sick neonates is a known risk factor for worse neurologic outcomes after ischemic brain injury [18]. Increase in cerebral metabolic rate in hyperthermic states is not desirable and may be related to poor postsurgical outcomes [3]. Neonates may be particularly vulnerable; even mild maternal pyrexia before delivery (≥37.6°C) is associated with ischemic-hypoxic injury [19]. Given the overall paucity of evidence on this topic, future research is necessary to examine the clinical implications of postoperative hyperthermia in infants, as its prevalence increases with expanding utilization of active warming measures in the perioperative period. Based on the frequency estimates presented here, it is likely that such a study will require analysis of data from multiple centers to achieve adequate power.

### 4.3. Practice Changes

Both hypothermia and hyperthermia were less likely with a procedure date since 2010, reflecting the effectiveness of two large scale interventions targeting postoperative hypothermia, namely, the introduction of closed-top incubators for intrahospital transports of neonates and sick infants and the rigorous implementation of intraoperative convective warming in an effort to increase compliance with SCIP-INF-10. This measure is one of the Centers of Medicare and Medicaid Services (CMS) and Physicians Quality Reporting Initiative endorsed quality indicators that are part of the Surgical Care Improvement Project (SCIP). Compliance with SCIP-INF-10 requires either a postoperative temperature of at least 36°C or documented use of intraoperative warming [20, 21]. At our institution, internally published reports on “SCIP-INF-10 noncompliance” were generated since spring 2010, leading to the adoption of almost universal use of warming blankets (see Figure 1). Because both the use of incubators and intraoperative warming blankets are expected to decrease hypothermia, this finding was anticipated, but the trend seemed to start even earlier than 2010. On the other hand, more aggressive use of warming devices may introduce the potential for iatrogenic overheating of infants, and we did see an upward trend in postoperative hyperthermia rates in the later years of the study period. Currently, the literature is relatively silent on unintended consequences of SCIP measures, but a recent multicenter study on effectiveness of a novel forced-air warming system in infants actually did show hyperthermia after 120 min of surgery (mean temperature of 37.5°C; range 36.1–38.4; *n* = 22) [16].

### 4.4. Incubators for Transport

From the perspective of the anesthesiologist, patient transport in a closed incubator does raise important safety concerns surrounding immediate access to the patient and, most critically, the airway. It is therefore not surprising that the introduction of closed-top incubators at our institution was met with skepticism by anesthesiologists. There is a paucity of evidence to support the effectiveness of incubators in reducing hypothermia for the patient transfer setting. One study of neonate transports from the delivery unit to the ICU did not show a clear benefit of incubators over radiant warmers in wrapped infants [22]. To the authors' knowledge, there are no reported studies addressing temperature homeostasis during postoperative transports. Notably, we did see a small but consistent increase in body temperature between end of surgery and arrival at the ICU. This effect may be a result of cessation of temperature loss by radiation once the infants are covered with blankets and placed in a warmed closed incubator. However, the present study cannot answer the question if the use of closed-top incubators for patient transport is causally related to the increasing percentage of patients with normothermia upon arrival at the ICU during the study period. We cannot separate those effects from the more aggressive use of intraoperative warming over the course of the study period. Still, closed-top incubators may have the greatest value in patients with postoperative hypothermia, even though the intraoperative drop in their body temperature was not offset by the modest increase during transfer to the ICU. Hyperthermic patients, on the other hand, experienced a relatively larger rise in temperature intraoperatively and during transport, suggesting that transport measures geared at heat conservation may exacerbate postoperative hyperthermia in patients who manifest significant intraoperative temperature increases. The recent rise in incidence of hyperthermia (albeit not statistically significant) at our institution may indicate growing utilization of closed-top incubators for postoperative patient transfers since their introduction in 2010.

### 4.5. Limitations

Limitations of the study lie in the retrospective design, which precludes making causal conclusions about the identified associations. Furthermore, the collected data include heterogeneous procedures, both surgical and nonsurgical, ranging from short to prolonged. Careful statistical analysis as described above was performed to reduce the possibility of confounders. Sample size limitations precluded addition of more variables in the models to prevent overfitting. Therefore, we admit the potential for further confounders not addressed in our models, including amount of volume administration or exposure to blood products. Finally, postoperative temperature values were obtained axillary, which may be considered less accurate than core temperature measurements. While this is likely true for the intraoperative environment due to exposure of the axilla to opposing forces such as either convective warming or room air, the axillary route is a widely accepted standard for measuring body temperature in infants in the intensive care unit [23].

## 5. Conclusions

At the authors' institution, there has been a significant improvement in achieving postoperative normothermia in critically ill infants during the study period, likely due to greater utilization of multimodal temperature conservation techniques resulting in fewer cases of hypothermia. Persistently high rates of postoperative hyperthermia suggest the need to carefully titrate active warming of patients to avoid unintended overheating.

## Figures and Tables

**Table 1:** Univariate analysis: characteristics of the study population, stratified by postoperative temperature. Hypothermia: postoperative temperature < 36.0°C; hyperthermia: postoperative temperature > 37.5°C.

	*N*	Hypothermia (*n* = 216) Mean (SD^1^) or *N* (%)	Normothermia (*n* = 1922) Mean (SD) or *N* (%)	Hyperthermia (*n* = 212) Mean (SD) or *N* (%)	*p* ^2^
Age (days)	2350	76 (82.26)	95 (97.58)	74 (91.11)	<0.01
Weight (kg)	2206	3.79 (1.88)	4.27 (2.21)	3.59 (2.02)	<0.01
Anesthesia duration (min)	2350	171 (103.37)	189 (112.53)	219 (112.53)	<0.01
Preoperative temperature (°C)	1555	36.64 (0.46)	36.69 (0.54)	36.79 (0.50)	0.10
Highest intraop. temperature (°C)	2030	36.27 (1.17)	37.07 (0.95)	37.89 (0.82)	<0.01
Last intraop. temperature (°C)	1787	35.05 (1.57)	36.29 (1.42)	36.96 (1.69)	<0.01
Postoperative temperature (°C)	2350	35.43 (0.61)	36.65 (0.41)	38.01 (0.42)	<0.01
Male gender	2350	117 (54%)	1128 (59%)	128 (60%)	0.37
ASA^3^ 4, 5	2350	95 (44%)	706 (37%)	91 (43%)	0.03
Emergent procedure	2350	42 (19%)	315 (16%)	47 (22%)	0.07
Premature birth (<37 weeks EGA^4^)	2218	110 (51%)	909 (47%)	110 (52%)	0.37
Postdate birth (>40 weeks EGA)	2218	2 (<1%)	14 (<1%)	2 (<1%)	0.91
Core temperature intraoperative monitoring^5^	2082	100 (46%)	1021 (53%)	146 (69%)	<0.01
Monitoring site					
(i) Skin		73 (34%)	682 (35%)	58 (27%)	
(ii) Axillary		0	2 (<1%)	0	
(iii) Nasal		68 (32%)	655 (34%)	90 (43%)	
(iv) Esophageal		30 (14%)	318 (17%)	50 (24%)	
(v) Bladder/rectal		1 (<1%)	45 (2%)	6 (3%)	
(vi) Oral/oropharyngeal		1 (<1%)	3 (<1%)	0	
Use of convective warming	2350	123 (57%)	1533 (80%)	196 (93%)	<0.01
Case date since 2010	2350	88 (41%)	1409 (73%)	116 (55%)	<0.01
Procedural service	2347				<0.01
(i) General surgery		93 (43%)	870 (45%)	132 (62%)	
(ii) ENT^6^		57 (26%)	361 (19%)	19 (9%)	
(iii) Neurosurgery		22 (10%)	230 (12%)	31 (15%)	
(iv) Other surgical service		39 (18%)	363 (19%)	22 (10%)	
(v) Nonsurgical service		5 (2%)	94 (5%)	8 (4%)	

^1^SD: standard deviation. ^2^ *p* values were calculated using Pearson chi-square test for categorical values and *t*-test or Kruskal-Wallis test for continuous variables. ^3^ASA: American Society of Anesthesiologists Physical Status classification. ^4^EGA: estimated gestational age. ^5^Core temperature includes esophageal, oral, oropharyngeal, nasal, bladder, and rectal temperature monitoring sites. ^6^ENT: Ear-Nose-Throat surgery.

**Table 2:** Logistic regression analysis for hypothermia compared to normothermia.

	Odds ratio	95% CI	*p*
Gender	1.39	0.905–2.120	0.13
Weight (kg)	0.89	0.784–0.999	0.05
Prematurity < 37 weeks	0.97	0.611–1.523	0.88
Postdate > 40 weeks	2.21	0.244–20.01	0.48
ASA status	1.71	1.109–2.623	0.02
Emergent procedure	1.23	0.709–2.135	0.46
Intraoperative temperature monitoring site	1.09	0.976–1.206	0.13
Intraoperative temperature change (°C)^1^	0.96	0.817–1.126	0.61
Anesthesia duration (min)	1.00	0.999–1.003	0.20
Convective warmer use	0.30	0.177–0.513	<0.001
Case date in 2010 or later	0.24	0.157–0.378	<0.001

^1^Intraoperative temperature change was calculated as the difference between the last intraoperative temperature value and the preoperative temperature.

**Table 3:** Logistic regression analysis for hyperthermia compared to normothermia.

	Odds ratio	95% CI	*p*
Gender	1.13	0.777–1.637	0.53
Weight (kg)	0.85	0.762–0.952	0.01
Prematurity < 37 weeks	0.81	0.544–1.213	0.31
Postdate > 40 weeks	2.42	0.474–12.31	0.29
ASA status	1.19	0.812–1.731	0.38
Emergent procedure	1.30	0.817–2.063	0.27
Intraoperative temperature monitoring site	1.00	0.906–1.094	0.92
Intraoperative temperature change (°C)^1^	1.35	1.217–1.488	<0.001
Anesthesia duration (min)	1.00	1.000–1.003	0.09
Convective warmer use	2.61	1.079–6.317	0.03
Case date in 2010 or later	0.33	0.221–0.502	<0.001

^1^Intraoperative temperature change was calculated as the difference between the last intraoperative temperature value and the preoperative temperature.

## Abbreviations

AIMS: Anesthesia Information Management System
ASA: American Society of Anesthesiologists
CI: Confidence interval
CMS: Centers of Medicare and Medicaid Services
ICU: Intensive care unit
IRB: Institutional review board
NICU: Neonatal intensive care unit
OR: Odds ratio
SCIP: Surgical Care Improvement Project
SD: Standard deviation.

## References

[1] Kurz A., Sessler D. I., Lenhardt R. (1996). Perioperative normothermia to reduce the incidence of surgical-wound infection and shorten hospitalization. Study of Wound Infection and Temperature Group. *The New England Journal of Medicine*.

[2] Morehouse D., Williams L., Lloyd C. (2014). Perioperative hypothermia in NICU infants: its occurrence and impact on infant outcomes. *Advances in Neonatal Care*.

[3] McCann M. E., Schouten A. N. J., Dobija N. (2014). Infantile postoperative encephalopathy: perioperative factors as a cause for concern. *Pediatrics*.

[4] Miller S. S., Lee H. C., Gould J. B. (2011). Hypothermia in very low birth weight infants: distribution, risk factors and outcomes. *Journal of Perinatology*.

[5] Galante D. (2007). Intraoperative hypothermia. Relation between general and regional anesthesia, upper- and lower-body warming: What strategies in pediatric anesthesia?. *Paediatric Anaesthesia*.

[6] Kim P., Taghon T., Fetzer M., Tobias J. D. (2013). Perioperative hypothermia in the pediatric population: a quality improvement project. *American Journal of Medical Quality*.

[7] Mathis M. R., Haydar B., Taylor E. L. (2013). Failure of the laryngeal mask airway unique™ and classic™ in the pediatric surgical patient: a study of clinical predictors and outcomes. *Anesthesiology*.

[8] Shah G. S., Christensen R. E., Wagner D. S., Pearce B. K., Sweeney J., Tait A. R. (2014). Retrospective evaluation of antimicrobial prophylaxis in prevention of surgical site infection in the pediatric population. *Paediatric Anaesthesia*.

[9] Harper C. M., Andrzejowski J. C., Alexander R. (2008). NICE and warm. *British Journal of Anaesthesia*.

[10] Hopf H. W. (2015). Perioperative temperature management: time for a new standard of care?. *Anesthesiology*.

[11] Mackowiak P. A., Wasserman S. S., Levine M. M. (1992). A critical appraisal of 98.6°F, the upper limit of the normal body temperature, and other legacies of Carl Reinhold August Wunderlich. *The Journal of the American Medical Association*.

[12] Narayan M., Medinilla S. P. (2013). Fever in the postoperative patient. *Emergency Medicine Clinics of North America*.

[13] Sessler D. I., Lee K. A., McGuire J. (1991). Isoflurane anesthesia and circadian temperature cycles in humans. *Anesthesiology*.

[14] Aoki K., Stephens D. P., Johnson J. M. (2001). Diurnal variation in cutaneous vasodilator and vasoconstrictor systems during heat stress. *American Journal of Physiology—Regulatory Integrative and Comparative Physiology*.

[15] Sessler D. I. (2013). Forced-air warming in infants and children. *Paediatric Anaesthesia*.

[16] Witt L., Dennhardt N., Eich C. (2013). Prevention of intraoperative hypothermia in neonates and infants: results of a prospective multicenter observational study with a new forced-air warming system with increased warm air flow. *Paediatric Anaesthesia*.

[17] Sun Z., Honar H., Sessler D. I. (2015). Intraoperative core temperature patterns, transfusion requirement, and hospital duration in patients warmed with forced air. *Anesthesiology*.

[18] Cotten C. M., Shankaran S. (2010). Hypothermia for hypoxic-ischemic encephalopathy. *Expert Review of Obstetrics and Gynecology*.

[19] Shankaran S., Laptook A. R., Ehrenkranz R. A. (2005). Whole-body hypothermia for neonates with hypoxic-ischemic encephalopathy. *The New England Journal of Medicine*.

[20] Surgical Care Improvement Project Core Measure Set. http://www.jointcommission.org/assets/1/6/Surgical%20Care%20Improvement%20Project.pdf.

[21] Lehtinen S. J., Onicescu G., Kuhn K. M., Cole D. J., Esnaola N. F. (2010). Normothermia to prevent surgical site infections after gastrointestinal surgery: holy grail or false idol?. *Transactions of the Meeting of the American Surgical Association*.

[22] Meyer M. P., Bold G. T. (2007). Admission temperatures following radiant warmer or incubator transport for preterm infants <28 weeks: a randomised study. *Archives of Disease in Childhood: Fetal and Neonatal Edition*.

[23] Robertson-Smith J., McCaffrey F. T., Sayers R., Williams S., Taylor B. J. (2015). A comparison of mid-forehead and axillary temperatures in newborn intensive care. *Journal of Perinatology*.

